# Discovery of Lanama Virus, a Distinct Member of Species *Kunsagivirus C* (*Picornavirales*: *Picornaviridae*), in Wild Vervet Monkeys (*Chlorocebus pygerythrus*)

**DOI:** 10.3390/v12121436

**Published:** 2020-12-14

**Authors:** Jens H. Kuhn, Samuel D. Sibley, Colin A. Chapman, Nick J. Knowles, Michael Lauck, Joshua C. Johnson, Cristine Campos Lawson, Matthew G. Lackemeyer, Kim Valenta, Patrick Omeja, Peter B. Jahrling, David H. O’Connor, Tony L. Goldberg

**Affiliations:** 1 Integrated Research Facility at Fort Detrick, National Institute of Allergy and Infectious Diseases, National Institutes of Health, Frederick, MD 21702, USA; joshua.carris.johnson@gmail.com (J.C.J.); cristine.c.lawson.civ@mail.mil (C.C.L.); matthew.lackemeyer@nih.gov (M.G.L.); jahrlingp@niaid.nih.gov (P.B.J.); 2 Department of Pathobiological Sciences, School of Veterinary Medicine, University of Wisconsin-Madison, Madison, WI 53706, USA; sdsibley@gmail.com; 3 Department of Anthropology, Center for the Advanced Study of Human Paleobiology, The George Washington University, Washington, DC 20052, USA; Colin.Chapman.Research@gmail.com; 4 School of Life Sciences, Pietermaritzburg Campus, University of KwaZulu-Natal, Scottsville 3209, South Africa; 5 Shaanxi Key Laboratory for Animal Conservation, School of Life Sciences, Northwest University, Xi’an 710069, China; 6 Makerere University Biological Field Station, P.O. Box 409, Fort Portal, Uganda; omejap@yahoo.com; 7 The Pirbright Institute, Pirbright, Woking, Surrey GU24 0NF, UK; nick.knowles@pirbright.ac.uk; 8 Department of Pathology and Laboratory Medicine, University of Wisconsin-Madison, Madison, WI 53705, USA; michaellauck@gmail.com (M.L.); dhoconno@wisc.edu (D.H.O.); 9 Department of Anthropology, University of Florida, Gainesville, FL 32603, USA; valentakim@gmail.com; 10 Wisconsin National Primate Research Center, Madison, WI 53715, USA

**Keywords:** *Chlorocebus pygerythrus*, vervet, kunsagivirus, lanama virus, LNMV, nonhuman primate, *Picornavirales*, *Picornaviridae*

## Abstract

We report the discovery and sequence-based molecular characterization of a novel virus, lanama virus (LNMV), in blood samples obtained from two wild vervet monkeys (*Chlorocebus pygerythrus*), sampled near Lake Nabugabo, Masaka District, Uganda. Sequencing of the complete viral genomes and subsequent phylogenetic analysis identified LNMV as a distinct member of species *Kunsagivirus C*, in the undercharacterized picornavirid genus *Kunsagivirus*.

## 1. Introduction

*Picornavirales* (*Pisoniviricetes*: *Pisuviricota* [1]) is the largest and most diverse viral order of positive-sense RNA viruses. The picornaviral family *Picornaviridae* alone includes 63 genera for viruses infecting hosts from all major vertebrate lineages. Among these viruses are significant pathogens, such as poliovirus, hepatitis A virus, and foot-and-mouth disease virus [2]. The diversity of viruses in this clade is likely much higher than currently recognized [3,4,5,6].

In 2013, a novel picornavirus was discovered in feces of a wild European roller (*Coracias garrulus* Linnaeus, 1758), a carnivorous migratory bird [7]. This virus is the founding member of novel picornavirid genus *Kunsagivirus* (after Kunság, a historical region in Hungary where the migratory bird was sampled) [7]. The virus was first named “greplavirus A” [7] and then kunsagivirus A1, and it was assigned to species *Kunsagivirus A* [2]. Since then, two other kunsagiviruses have been described. In 2017, “bat kunsagivirus” (now known as kunsagivirus B1) was identified in feces of wild African straw-colored fruit bats (*Eidolon helvum* Kerr, 1792), sampled in Southwest Cameroon [6]. In the same year, bakunsa virus (BKUV, also known as kunsagivirus C1) was discovered by metagenomic sequencing in blood from a wild yellow baboon (*Papio cynocephalus* Linnaeus, 1766), sampled in Tanzania in 1986 [8]. Today, these two viruses are classified into species *Kunsagivirus B* and *Kunsagivirus C*, respectively [2]. Kunsagiviruses have yet to be isolated in culture, and the morphologies of their virions remain unknown [2].

Here, we report the discovery and sequence-based molecular characterization of a novel kunsagivirus, lanama virus (LNMV), in blood samples obtained from two wild vervet monkeys (*Chlorocebus pygerythrus* F. Cuvier, 1821), sampled near Lake Nabugabo, Uganda.

## 2. Materials and Methods

One male and two female apparently healthy wild adult vervet monkeys were sampled for virus discovery at Lake Nabugabo, a satellite lake of Lake Victoria, in Masaka District, Central Region, Uganda. The sampled monkeys were members of a habituated group of vervet monkeys, studied since May 2011 [9]. (The current composition of genus *Chlorocebus* is disputed, with monkeys found in this region of Uganda being variously identified as vervet monkeys (*C. pygerythrus*) or tantalus monkeys (*C. tantalus* Ogilby, 1841) [10,11]. In keeping with the current taxonomic standards in the literature, we elected to retain the commonly used taxonomy and hence refer to the sample monkeys as vervet monkeys.) The monkeys were anesthetized, and blood was sampled using previously described field methods [12,13]. Blood was separated in the field by centrifugation (15 min, 3000× *g*) and stored in liquid nitrogen until processing. RNA was extracted and converted to cDNA, which was sequenced on an Illumina MiSeq instrument according to previously published methods [14]. Briefly, RNA was extracted from blood plasma using the QIAamp Viral RNA Mini Kit (Qiagen, Hilden, Germany), RNA was converted to cDNA using random hexamers and the Superscript IV system (Thermo Fisher, Waltham, MA, USA), and libraries were prepared for sequencing on a MiSeq instrument (V2 chemistry, 300 cycle kit; Illumina, San Diego, CA, USA), using the Nextera XT DNA sample preparation kit (Illumina, San Diego, CA, USA). Sequence reads were quality-trimmed (discarding sequences with quality scores < q30 and lengths < 75 bases), and contiguous sequences (contigs) were assembled de novo using CLC Genomics Workbench version 11.1 (CLC Bio, Aarhus, Denmark). Sequence reads and assembled contigs were then compared at the nucleotide and deduced amino-acid sequence level to sequences in GenBank using the BLASTn and BLASTx homology searching algorithms, respectively [15].

Translated amino-acid sequences were aligned using Clustal W software [16] as implemented in BioEdit 7.2.5 [17]. The evolutionary history of the newly discovered virus genomes was inferred by using the maximum-likelihood method based on the Le and Gascuel (2008) amino-acid substitution model [18]. Initial phylogenetic tree(s) for the heuristic search were obtained automatically by applying Neighbor-Join and BioNJ algorithms to a matrix of pairwise distances estimated using a JTT model and then selecting the topology with superior log likelihood value. A discrete Gamma distribution (+G) was used to model evolutionary rate differences among sites (five categories (+G; parameter = 2.6468)). The rate-variation model allowed for some sites (5.87%) to be evolutionarily invariable (+I). The analysis involved 53 amino-acid sequences. All positions with less than 95% site coverage were eliminated, i.e., fewer than 5% alignment gaps, missing data, and ambiguous bases were allowed at any position. A total of 565 positions were included in the final dataset. Evolutionary analyses were conducted in MEGA7 [19].

Virus isolation was attempted in Vero E6 cells (American Type Culture Collection (ATCC), Manassas, VA, USA; CRL-1586), seeded into wells of a 96-well plate according to ATCC culture recommendations. An amount of 50 µL of primate plasma or mock plasma (medium containing 10% heat-inactivated fetal bovine serum (Thermo Fisher Scientific, Waltham, MA, USA) was added to each well and incubated for 1 h at 37 °C and 5% CO_2_). The initial inocula were recovered and sequentially added in the same manner to human cervical carcinoma HeLa cells (ATCC CCL-2) and baby hamster kidney BHK-21 cells (ATCC CCL-10). Next, 200 µL of prewarmed media was added to each well, and cells were incubated and observed daily for cytopathic effect for 5 days. Media were removed from cells, replaced with 200 µL per well of fresh media, and cells were lysed by three freeze/thaw rounds at −80 °C. The resulting lysates were collected and pooled with the respective cell supernatants. For Passage 2, 300 µL of pooled supernatants and lysates from each sample condition was added to fresh cells in six-well plates under gentle agitation. Then, 2 mL of prewarmed media was added to each well, and cells were incubated and observed daily for cytopathic effect. On Day 0, Day 3, and Day 5 after inoculation, 200 µL of media was collected from each well and mixed with 600 µL of TRIzol LS (Thermo Fisher Scientific) for virus inactivation and downstream sequencing analysis. Removed volumes were replaced with prewarmed media.

## 3. Results

**Figure 1:**
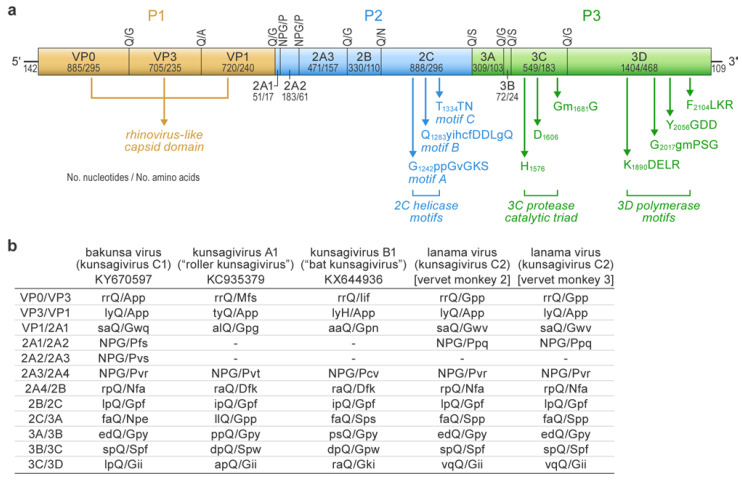
Lanama virus genome organization. (**a**) The cartoon, drawn to scale, outlines the genome of lanama virus (vervet monkey 2), demonstrating the typical nonsegmented, linear, single-stranded, positive-sense genome of picornavirids that encodes a single polyprotein, which is post-translationally proteolytically cleaved into polyproteins P1–P3. Picornavirid-typical protein motifs are indicated by vertical arrows. (**b**). The polyprotein is cleaved into structural and nonstructural proteins at conserved cleavage sites as determined by alignment with the polyproteins of other kunsagiviruses (see Figure S1). The cleaved residues are shown in upper case, whereas those preceding and following are shown in lower case, except for the NPG↓P ribosome-skipping motif (shown in upper case).

**Figure 2:**
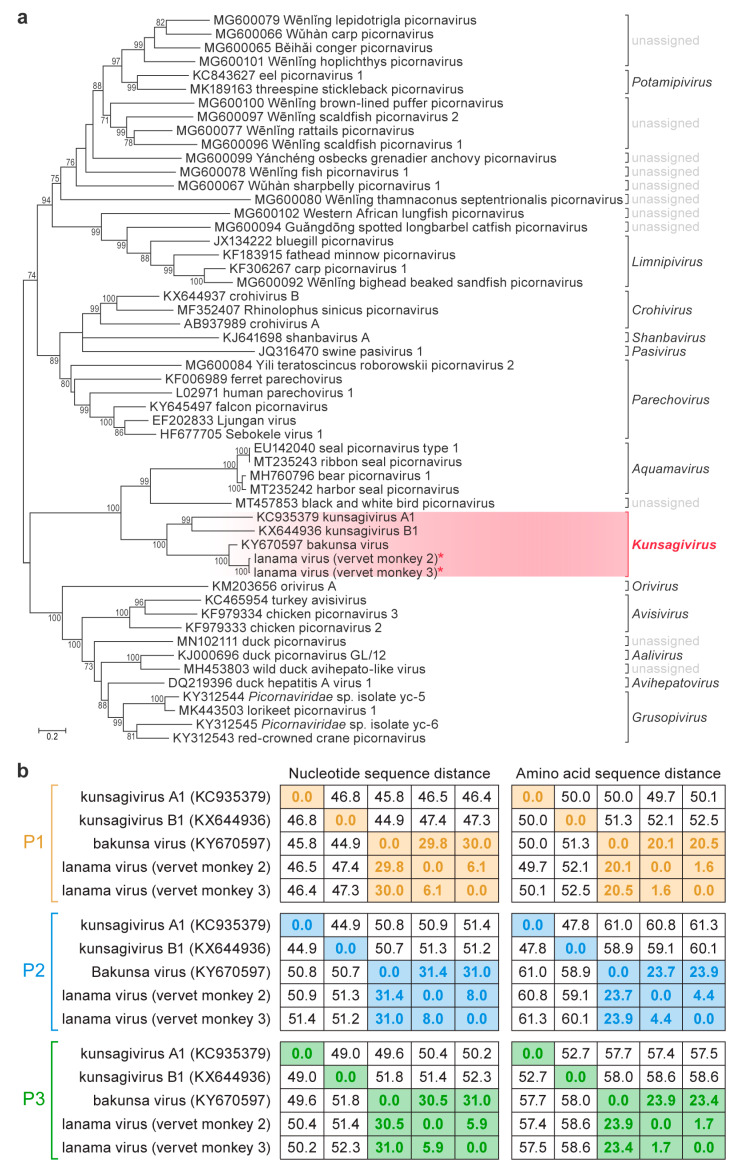
Phylogenetic analysis of kunsagiviruses. (**a**) Molecular phylogenetic analysis of *Picornaviridae* Supergroup 4 genera by the maximum-likelihood method. The tree with the highest log likelihood (−48,587.18) is shown. The percentage of trees in which the associated taxa cluster together is shown next to the branches. The tree is drawn to scale, with branch lengths measured in the number of substitutions per site. Each branch is labelled with the GenBank accession number associated with the analyzed sequence followed by the current virus name. (**b**) Nucleotide and amino-acid sequence distances (p-dist) of kunsagivirus precursor proteins P1–P3 were determined using MEGA7 [19].

Deep sequencing yielded, after quality trimming, 4,203,737, 3,185,890, and 5,375,995 sequences of 155, 154, and 164 nucleotide average length for vervet monkeys 1, 2, and 3, respectively. Analysis of these sequences revealed the presence of a novel picornavirid in the blood of the two female vervet monkeys (vervet monkeys 2 and 3), whereas the male vervet monkey (vervet monkey 1) appeared to be uninfected. There were also putative hits to varied retrovirids, small circular DNA viruses, and hepadnavirids, but these were not investigated further due to ambiguous taxonomic assignments and low depth of coverage. In silico analyses enabled the assembly of near-complete picornavirid genomes (with approximately 390 nucleotides missing from the 5′ end of each genome due to depletion of biological material) from both individuals (GenBank #MW218667 and #MW218668), with 337 and 134 reads from vervet monkeys 2 and 3, respectively, mapping to the picornavirid. Comparison of the obtained genome sequences to published picornavirid genomes enabled the identification of picornavirid-typical cleavage sites in the encoded polyprotein and, thus, the description of the genomic organization (Figure 1). Sequence alignments (Figures S1 and S2) and phylogenetic analyses (Figure 2) that comprise all currently known kunsagiviruses (A1, B1, and BKUV) indicated that the two viruses from the two female vervet monkeys are closely related to each other (Figure 2) and represent isolates of a novel kunsagivirus. We named the new virus lanama virus (LNMV) after the location where it was discovered (Lake Nabugabo, Masaka District). Kunsagiviruses appear to encode two to four 2A proteins [6,7,8]. The last encoded 2A protein in each virus possesses a typical 3C cleavage site (Q/G), separating it from the 2B protein. All other 2A proteins are separated from adjacent proteins via a ribosome-skipping mechanism involving an NPG↓P sequence motif [20]. Although the two lanama virus genome sequences are most closely related to BKUV (*Kunsagivirus C*), they appear to lack one of the 2A proteins (possibly 2A2) (Figure S2). The established species demarcation criteria for *Kunsagivirus* [2] and the VP1 p-dist values of >33% nucleotides and >21% amino acids suggest that LNMV is a distinct member of species *Kunsagivirus C*, representing a distinct genotype (kunsagivirus C2). All virus isolation attempts were unsuccessful.

## 4. Discussion

Kunsagiviruses A1 and B1 were discovered in feces samples of birds and bats, respectively [6,7]. Thus, it is possible that both viruses infect hosts in the diet of these vertebrates rather than infecting the vertebrates themselves. However, the discovery of BUKV and now LNMV in blood of apparently healthy nonhuman primates indicates that kunsagiviruses indeed are vertebrate viruses and further supports the notion that kunsagiviruses are widely geographically distributed (in Africa and Europe) and have broad host ranges (birds, bats, nonhuman primates, and likely other animals). Consequently, further studies should be performed to better define kunsagivirus diversity, host range, and pathogenic potential.

## Supplementary Materials

The following are available online at https://www.mdpi.com/1999-4915/12/12/1436/s1, Figure S1: Kunsagivirus polyprotein alignment using available complete kunsagivirus sequences. Figure S2: Comparison of the four types of kunsagivirus 2A proteins using available complete kunsagivirus sequences.
