# Supplementary figures and images for: Discovery of Lanama Virus, a Distinct Member of Species Kunsagivirus C (Picornavirales: Picornaviridae), in Wild Vervet Monkeys (Chlorocebus pygerythrus)
Source: Viruses. 2020 Dec 14;12(12):1436. doi: 10.3390/v12121436 (PMC7764893; doi:10.3390/v12121436)

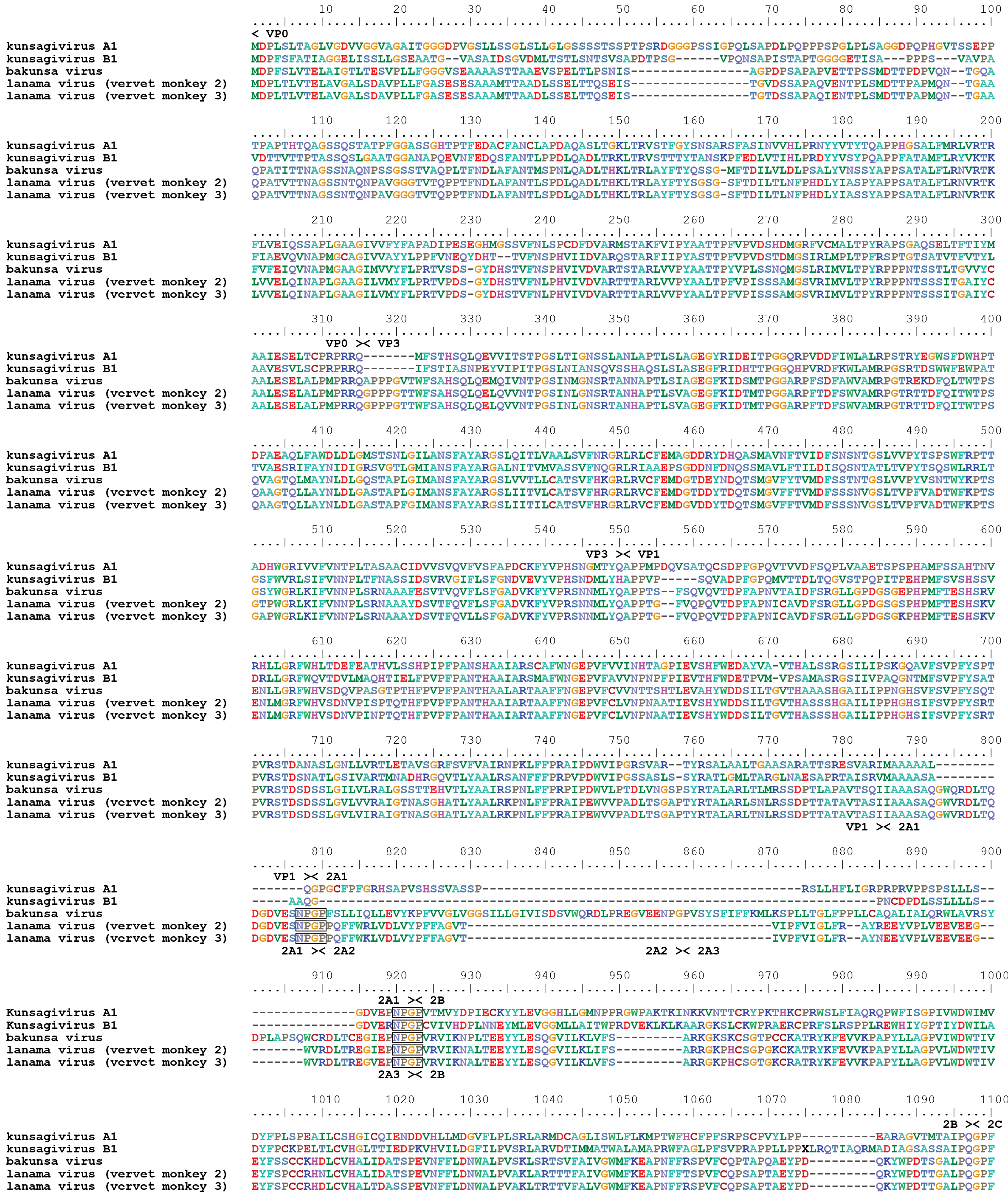

Supplement: Supplementary file 1 [file viruses-12-01436-s001.zip › Figure S1-1.tif]

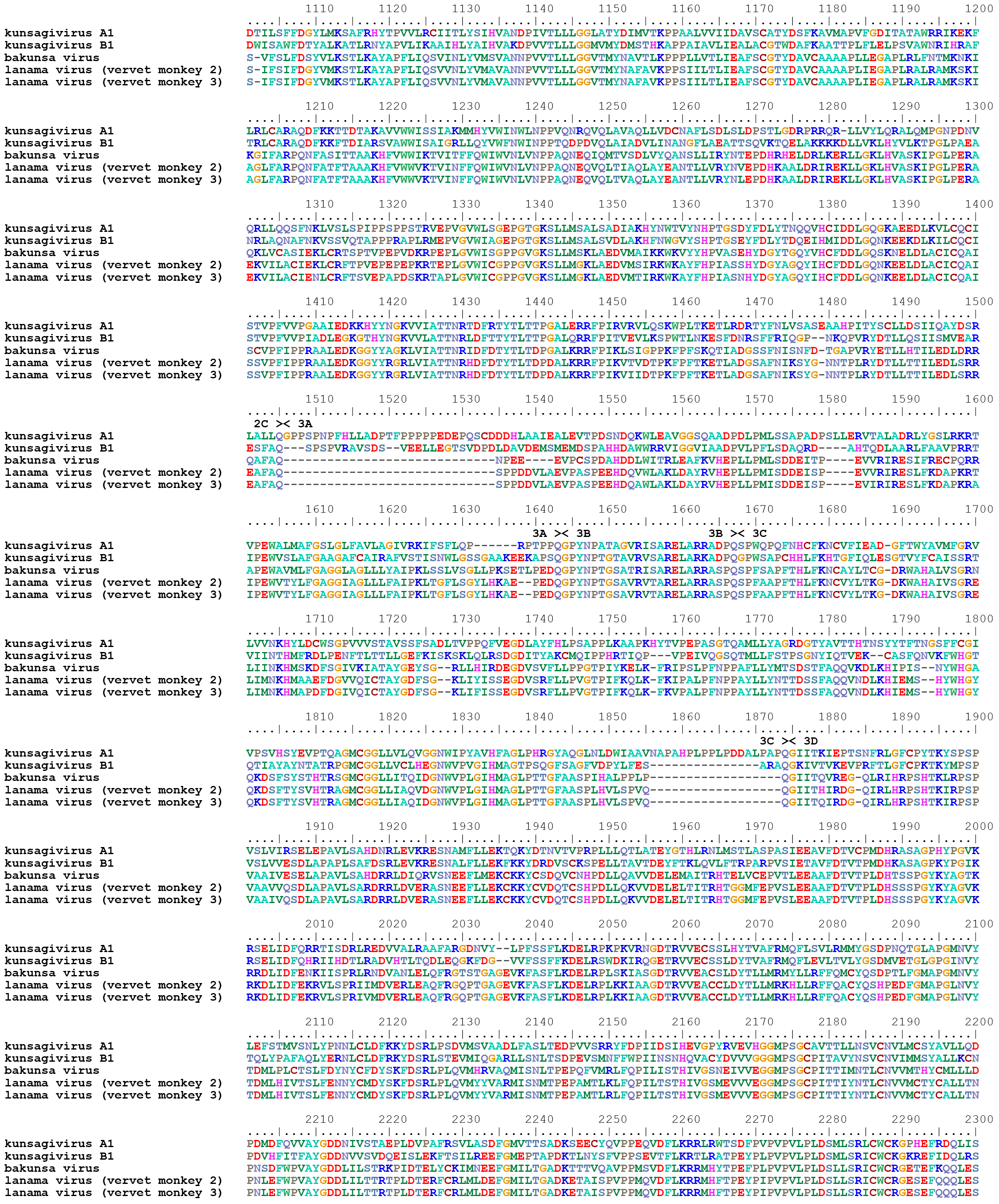

Supplement: Supplementary file 1 [file viruses-12-01436-s001.zip › Figure S1-2.tif]

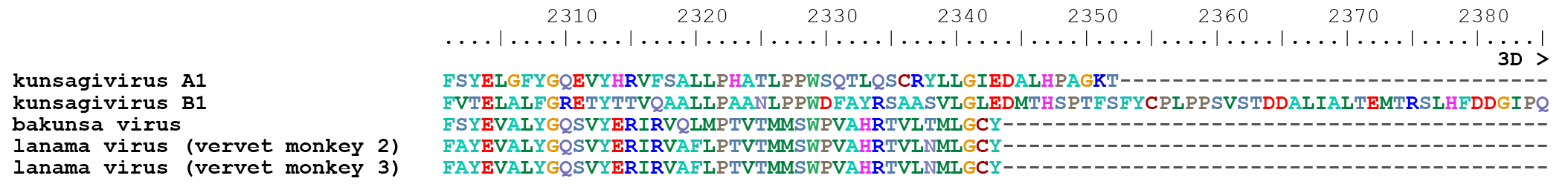

Supplement: Supplementary file 1 [file viruses-12-01436-s001.zip › Figure S1-3.tif]

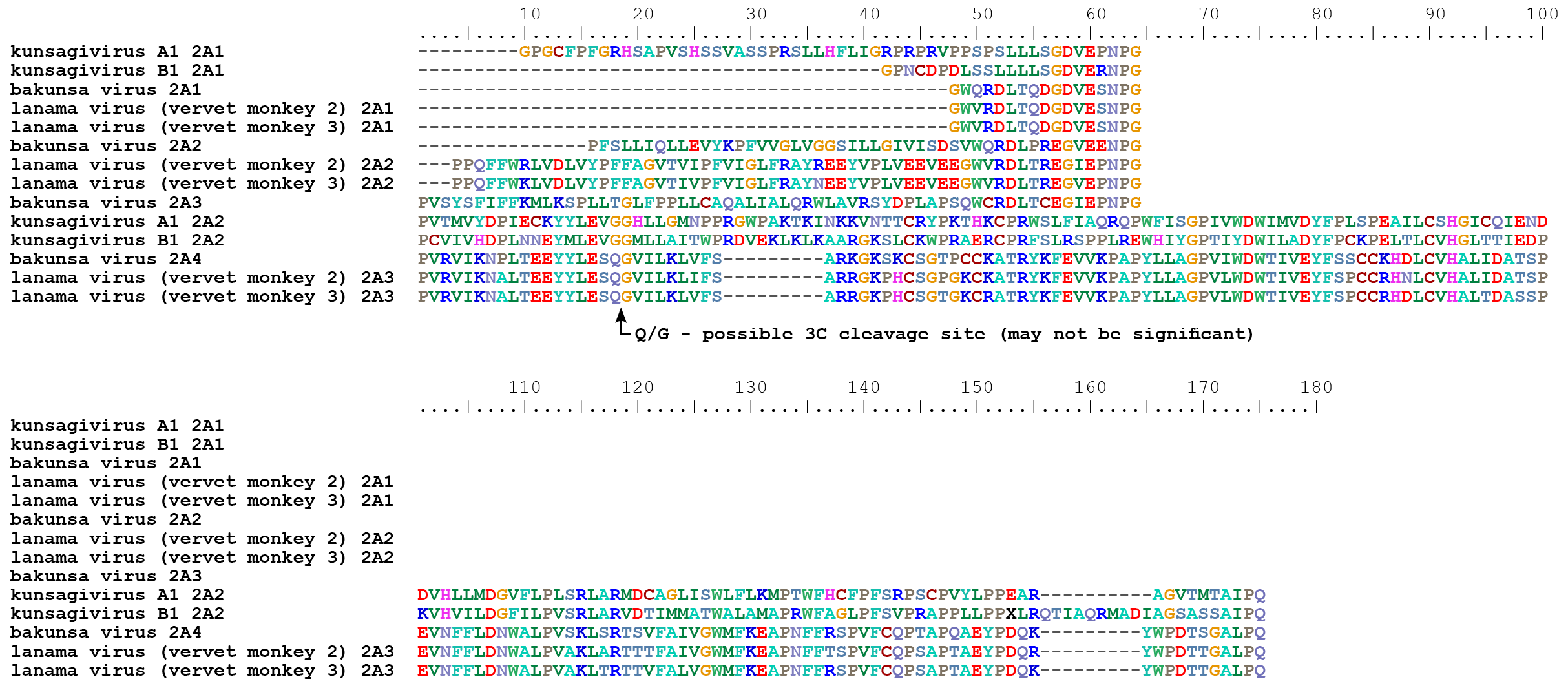

Supplement: Supplementary file 1 [file viruses-12-01436-s001.zip › Figure S2.tif]
